# Clinical and lateral cephalometric evaluation of class II elastic therapy in skeletal class II malocclusion cases indicated for fixed functional appliance: An *in vivo* study

**DOI:** 10.6026/973206300223545

**Published:** 2026-06-30

**Authors:** Priyanshi Mahajan, Purva Joneja, Neelam Dhakar, Julius Mary, Rishabh Joneja, Priyanka jaiswal

**Affiliations:** 1 Department of Orthodontics and Dentofacial Orthopedics, Bhabha College of Dental Sciences, Bhopal, Madhya Pradesh, India; 2 Department of Orthodontics and Dentofacial Orthopedics, District Hospital, Chhindwara, Madhya Pradesh, India; 3 Department of Medicine, First Faculty of Medicine, Charles University, Prague, Czech Republic; 4 Department of Orthodontics and Dentofacial Orthopedics, Mahatma Gandhi District Hospital, Indore, Madhya Pradesh, India

**Keywords:** Class II malocclusion, elastic, cephalometrics, orthodontics

## Abstract

Management of skeletal Class II malocclusion remains a persistent orthodontic challenge, particularly in selecting an effective alternative to fixed functional appliances. Therefore, it is of interest to evaluate the clinical and cephalometric effects of Class II elastics in 10 patients with skeletal Class II malocclusion. Pre- and post-treatment cephalometric parameters, including SNA, SNB, ANB and overjet, were compared using paired t-test analysis. Data shows significant improvement in cephalometric angles and overjet reduction, with dentoalveolar changes being more pronounced than skeletal changes. Thus, we show Class II elastics as a clinically effective alternative in borderline skeletal Class II cases, with outcomes comparable to fixed functional therapy in selected patients.

## Background:

Malocclusion refers to any deviation from the ideal alignment and occlusal relationship of teeth and jaws, resulting in an abnormal contact between the maxillary and mandibular teeth when the jaws are closed [1]. It may involve irregularities in tooth position, arch relationship, or jaw relationship, affecting esthetics, function and oral health. Malocclusion can be classified based on angles classification Class I, Class II and Class III [2]. Class II malocclusion is one of the most prevalent orthodontic problems encountered in clinical practice, often characterized by mandibular retrusion, maxillary protrusion, or a combination of both. Various treatment modalities have been proposed, including growth modification using functional appliances, camouflage treatment using fixed appliances and orthognathic surgery in severe cases [3]. Fixed functional appliances are widely accepted for correcting skeletal discrepancies; however, they may be associated with patient discomfort, increased cost and compliance-independent mechanics. Alternatively, Class II elastics are commonly used due to their simplicity, cost-effectiveness and ease of application [4]. Despite their widespread use, the effectiveness of Class II elastics in patients ideally suited for fixed functional appliance therapy remains controversial. Most studies suggest that elastics primarily produce dento alveolar changes rather than true skeletal correction [5]. Therefore, it is of interest to evaluate the clinical and cephalometric effects of Class II elastics in skeletal Class II malocclusion patients and assess their viability as an alternative to fixed functional appliances.

## Methodology:

This prospective study was conducted in the Department of Orthodontics at Bhabha College of Dental Sciences, Bhopal, on a sample of 10 patients diagnosed with skeletal Class II malocclusion. Patients were selected according to predefined inclusion and exclusion criteria. The inclusion criteria comprised skeletal Class II malocclusion with a minimum half Class II molar relationship treated on a non-extraction basis, increased overjet, retrognathic mandible, complete permanent dentition with or without third molars, age between 14 and 25 years and good oral hygiene. Patients were excluded if they had undergone previous orthodontic treatment, were uncooperative, had any systemic disorder, Class I or Class III malocclusion, craniofacial anomalies, history of major facial trauma, were examined before initial leveling and alignment, or presented with severe skeletal discrepancies requiring surgical correction. The study was undertaken to evaluate the use of Class II elastics in the treatment of Class II malocclusion and to determine their principal skeletal, dental and soft-tissue effects. All patients were treated with fixed mechanotherapy using 0.022-inch slot MBT prescription brackets. After completion of leveling and alignment, Class II elastics were applied from the maxillary canine to the mandibular first molar. The study population demonstrated pre-treatment characteristics consistent with Class II malocclusion, including a mean ANB angle of 5.00°C, WITS appraisal of 3.50 ± 0.71 mm, SNA angle of 79.80 ± 0.79°C and SNB angle of 75.30 ± 0.48°C, indicating a relatively retrusive mandible. The vertical pattern was represented by a mean FMA of 19.50 ± 0.53°C and a saddle angle of 128.90 ± 0.88°C. Dental characteristics included mandibular incisor proclination and protrusion, with mean IMPA of 100.30 ± 1.06°C, L1-NB of 31.50 ± 1.65°C and U1-NA of 27.80 ± 1.14°C, along with an overjet of 4.70 ± 0.48 mm and an overbite of 4.60 ± 0.52 mm. Soft-tissue assessment revealed a convex profile, with a mean H-angle of 17.60 ± 0.70°C and lip step of 2.50 ± 0.53 mm. The mechanics of elastic wear were based on evidence from previously accepted studies. A 3/16-inch Class II elastic was used, with a mean force of 2.6 oz, as reported across five articles and force levels were measured with a tension gauge. In accordance with published evidence, elastics were used on rigid archwires, particularly 0.016 x 0.022-inch stainless steel wires, to minimize unfavorable effects such as clockwise rotation of the occlusal plane. Full-time wear was prescribed and in patients with deep overbite, reversed and accentuated curves of Spee were considered for vertical control. The duration of active elastic wear generally extended for at least 6 months, with an average treatment period of approximately 8.5 months for sagittal correction. Since Class II elastics are highly compliance-dependent, all patients were instructed regarding the importance of consistent wear and active retention was advised after sagittal correction to improve long-term stability. Lateral cephalograms were obtained before and after treatment and both skeletal and dental parameters were analyzed. The measured variables included SNA, SNB, ANB, FMA, WITS appraisal, saddle angle, lower anterior facial height, pogonion prominence, L1-NB, IMPA, U1-NA, overjet, overbite, lip step and H-angle. Statistical analysis was performed using the paired t-test to compare pre- and post-treatment values. In order to validate the outcomes of the present study, a high-quality systematic review synthesizing data from 11 clinical studies identified from PubMed, Scopus, Web of Science, Embase, Medline and Cochrane databases was used as a comparative framework. This review established the expected dentoalveolar and skeletal effects of Class II elastics, thereby enabling the results of the present study to be interpreted against the broader evidence base.

## Formula:

## Student 'T' Test:

The comparison of means between two independent groups for continuous variables was performed using the Independent Student's t-test (see PDF).

where the pooled standard deviation (Sp) is calculated as:(see PDF)

Where:

Sp = Pooled standard deviation

S_1_ = Standard deviation of Group 1

S_2_ = Standard deviation of Group 2

n_1_ = Number of samples in Group 1

n_2_ = Number of samples in Group 2

see PDF = Mean of Group 1

see PDF = Mean of Group 2

Degrees of Freedom (df):

df = n_1_ + n_2_ - 2

The significance of the difference between the groups was determined by the obtained p-value.

P < 0.001 = Highly Significant

P < 0.05 = Significant

P > 0.05 = Not Significant

## Results:

All the finding obtained were tabulated in Microsoft excel sheets. Statistical analysis was done using SPSS software version 26.0. Mean and standard deviation will be estimated in the sample for each study group. Mean values will be compared by using independent students "t" test for two group comparison. P<0.05 was considered as the level of significance. The cephalometric parameters of the participants were evaluated before and after treatment (Table 1). Descriptive statistics including mean, standard deviation, minimum and maximum values were calculated for all variables. Independent Student's t-test was used to determine the statistical significance between pre-treatment and post-treatment measurements. A p-value <0.05 was considered statistically significant and p < 0.001 was considered highly significant. All subjects demonstrated Class II malocclusion prior to treatment. The mean overjet was 4.70 ± 0.48 mm and the mean overbite was 4.60 ± 0.52 mm, indicating increased horizontal and vertical overlap of the incisors. Skeletal parameters showed a mean SNA angle of 79.80 ± 0.79°C, SNB angle of 75.30 ± 0.48°C and ANB angle of 5.00°C, confirming a skeletal Class II relationship. The FMA was 19.50 ± 0.53°C and the WITS appraisal averaged 3.50 ± 0.71 mm, suggesting a sagittal skeletal discrepancy. Dental measurements revealed a mean L1-NB angle of 31.50 ± 1.65°C, IMPA of 100.30 ± 1.06°C and U1-NA angle of 27.80 ± 1.14°C, indicating proclination of the incisors. Soft tissue parameters showed a mean lip step of 2.50 ± 0.53 mm and H-angle of 17.60 ± 0.70°C, indicating a convex soft tissue profile (Table 2). Following treatment, a considerable reduction in dental discrepancies was observed. The mean overjet decreased to 2.20 ± 0.42 mm and the mean overbite decreased to 2.30 ± 0.48 mm, indicating improvement in incisor relationship. Among skeletal parameters, the SNA angle remained unchanged (79.80 ± 0.79°C), suggesting no significant change in maxillary position. However, the SNB angle increased to 80.40 ± 0.52°C, reflecting forward positioning of the mandible. Consequently, the ANB angle decreased to 2.10 ± 0.32°C, indicating correction toward a Class I skeletal relationship. The FMA increased to 21.80 ± 1.14°C, while the WITS appraisal decreased to 1.50 ± 0.53 mm, demonstrating improvement in sagittal jaw relationships. Dental parameters also showed improvement with a reduction in L1-NB (27.10 ± 1.29°C), IMPA (94.70 ± 0.48°C) and U1-NA (22.20 ± 1.48°C). Soft tissue analysis revealed a decrease in lip step (1.00 ± 0.82 mm) and H-angle (13.00 ± 0.94°C), suggesting improvement in facial profile (Table 3). Comparison of pre-treatment and post-treatment measurements demonstrated significant improvements in several cephalometric parameters. The mean overjet reduced from 4.70 mm to 2.20 mm and the overbite decreased from 4.60 mm to 2.30 mm following treatment. Skeletal analysis showed an increase in SNB from 75.30°C to 80.40°C, while the ANB angle decreased from 5.00°C to 2.10°C, indicating correction of the skeletal Class II pattern. The WITS appraisal decreased from 3.50 mm to 1.50 mm, further confirming improvement in sagittal jaw relationships. Dental parameters showed favorable changes with reductions in L1-NB, IMPA and U1-NA, indicating normalization of incisor inclination. Soft tissue measurements also demonstrated improvement with reductions in lip step and H-angle (Table 4). Inferential statistical analysis using the independent Student's t-test revealed statistically highly significant differences between pre-treatment and post-treatment values for most parameters. Significant reductions were observed in overjet (p < 0.001) and overbite (p < 0.001). Skeletal parameters showed significant improvement with an increase in SNB (p < 0.001) and a reduction in ANB (p < 0.001). Similarly, FMA and WITS appraisal showed statistically significant changes (p < 0.001). Dental parameters including L1-NB, IMPA and U1-NA demonstrated highly significant reductions (p < 0.001), indicating improved incisor positioning. Soft tissue parameters such as lip step and H-angle also showed statistically significant improvement (p < 0.001). However, the SNA angle did not show a statistically significant change (p = 1.000), suggesting that the treatment primarily affected mandibular position rather than the maxilla (Table 5). Overall, the results demonstrated significant improvements in skeletal, dental and soft tissue parameters following treatment. The reduction in overjet, overbite, ANB angle, WITS appraisal and incisor proclination, along with the increase in SNB angle, indicates effective correction of the Class II malocclusion and improvement in facial profile. Table 5 shows Independent t test for comparison of the mean cephalometric values of the participants before and after treatment.

## Discussion:

The present study evaluated the cephalometric changes following treatment of skeletal Class II malocclusion. The significant reduction in overjet and overbite indicates effective correction of the anterior dental relationship and improved incisor positioning. Skeletal analysis revealed a significant increase in the SNB angle and reduction in ANB angle and WITS appraisal, suggesting improvement in mandibular position and sagittal jaw relationship. Dental parameters such as L1-NB, IMPA and U1-NA showed significant reduction, indicating improved inclination and alignment of incisors. Additionally, the decrease in lip step and H-angle suggests improvement in the soft tissue profile and facial esthetics.The result of this study aligns with the findings of Janson *et al.* (2023) [6] , which concluded that Class II elastics are effective tools for malocclusion correction, while many practitioners default to fixed functional appliances for mandibular retrusion. Our findings support the comparable effectiveness theory. Our protocol utilized 0.022-in slots, providing a different torque expression environment than the 0.018-in slots often cited in previous studies. However, the use of 0.016 x 0.022-inch stainless steel working wire proved stable enough to support the intermaxillary force of 3/16 elastics. The 8.5-month duration used in this study matches the average period suggested in the literature for obtaining correction [7]. This time frame appears to be a critical threshold for balancing tooth movement with the potential for the side effects, like occlusal plane rotation, often feared in long-term elastic use.The analysis of cephalometric parameters revealed significant improvements, with the mean overjet reducing from 4.70 mm to 2.20 mm post-treatment. Skeletal analysis indicated an increase in the S-N-B angle and a decrease in the A-N-B angle, signifying correction of the skeletal Class II relationship [8]. Additionally, the Wits appraisal decreased, further confirming improvement in the sagittal jaw relationship. Dental parameters also exhibited favorable changes, with reductions in L1-NB, IMPA and U1-NA, indicating normalization of incisor inclination. Soft tissue measurements also improved, with reductions in both the lip-to-teeth ratio [9, 10]. Overall, the results demonstrate that the treatment approach effectively corrected skeletal Class II malocclusion by improving mandibular position, dental relationships and facial profile.

## Conclusion:

Class II elastics are effective in patients aged 14-25 years with mild to moderate skeletal Class II malocclusion and mandibular retrognathism. When used with a 0.022-inch bracket system and 0.016 x 0.022-inch stainless steel working wire, they provide a predictable response and achieve satisfactory sagittal correction within approximately 8.5 months. Further longitudinal studies with 2-3 years of follow- up are required to determine the long-term stability of these effects and whether the outcomes remain distinct from, or eventually converge with, those of functional appliances.

## Figures and Tables

**Table 1:** Cephalometric parameters analyzed in the study

**Parameter Type**	**Parameter**	**Definition**
Skeletal	SNA (°C)	Angle formed by the intersection of lines from Sella (S) and Point A to Nasion (N), representing the anteroposterior position of the maxilla relative to the cranial base.
Skeletal	SNB (°C)	Angle formed by the intersection of lines from Sella (S) and Point B to Nasion (N), representing the anteroposterior position of the mandible relative to the cranial base.
Skeletal	ANB (°C)	Angle representing the relative anteroposterior relationship of the maxilla to the mandible, formed by lines from Points A and B to Nasion (N).
Skeletal	FMA (°C)	Frankfort mandibular plane angle measuring the vertical orientation of the mandible relative to the Frankfort horizontal plane.
Skeletal	WITS (mm)	Linear appraisal of the sagittal relationship between the maxilla and mandible, measured as the distance between perpendicular projections of Points A and B onto the functional occlusal plane.
Skeletal	Saddle angle (°C)	Cephalometric angle, typically N-S-Ar, used to evaluate the relationship between the anterior and posterior cranial base.
Skeletal	LAFH (mm)	Lower anterior facial height, defined as the linear distance from anterior nasal spine (ANS) to menton.
Skeletal	MAN/POG	Evaluates the prominence or advancement of the chin using Pogonion (Pg), the most anterior point on the bony chin.
Dental	L1-NB (°C)	Inclination or position of the mandibular central incisor relative to the Nasion-Point B line.
Dental	IMPA (°C)	Angle formed by the long axis of the mandibular incisor relative to the mandibular plane.
Dental	U1-NA (°C)	Inclination or position of the maxillary central incisor relative to the Nasion-Point A line.
Dental	Overjet (mm)	Horizontal distance between the incisal edges of the maxillary and mandibular central incisors, measured parallel to the functional occlusal plane.
Dental	Overbite (mm)	Vertical distance between the incisal edges of the maxillary and mandibular central incisors, measured perpendicular to the Frankfort plane.
Soft tissue	Lip step	Linear measurement evaluating the horizontal relationship between the upper and lower lips.
Soft tissue	H-angle	Holdaway soft-tissue angle used to evaluate the convexity of the soft-tissue profile.

**Table 2:** Mean cephalometric values of the participants before treatment

**Parameter**	**Mean**	**Standard Deviation**	**Minimum**	**Maximum**
Molar position	2	0	2	2
(mm)				
Overjet	4.7	0.48	4	5
Overbite	4.6	0.52	4	5
SNA	79.8	0.79	79	81
SNB	75.3	0.48	75	76
ANB	5	0	5	5
FMA	19.5	0.53	19	20
WITS	3.5	0.71	2	4
Saddle	128.9	0.88	128	130
L1NB	31.5	1.65	30	34
IMPA	100.3	1.06	99	102
U1NA	27.8	1.14	26	29
Lip Step	2.5	0.53	2	3
H angle	17.6	0.7	16	18

**Table 3:** Mean cephalometric values of the participants after treatment

**Parameter**	**Mean**	**Standard Deviation**	**Minimum**	**Maximum**
Molar position (mm)	1	0	1	1
Overjet	2.2	0.42	2	3
Overbite	2.3	0.48	2	3
SNA	79.8	0.79	79	81
SNB	80.4	0.52	80	81
ANB	2.1	0.32	2	3
FMA	21.8	1.14	20	23
WITS	1.5	0.53	1	2
Saddle	125.1	0.88	123	126
L1NB	27.1	1.29	25	29
IMPA	94.7	0.48	94	95
U1NA	22.2	1.48	20	24
Lip Step	1	0.82	0	2
H angle	13	0.94	12	15

**Table 4:** Comparison of the mean cephalometric values of the participants before and after treatment

**Parameter**	**Pre Mean**	**Pre SD**	**Pre Min**	**Pre Max**	**Post Mean**	**Post SD**	**Post Min**	**Post Max**
Molar position (mm)	2	0	2	2	1	0	1	1
Overjet	4.7	0.48	4	5	2.2	0.42	2	3
Overbite	4.6	0.52	4	5	2.3	0.48	2	3
SNA	79.8	0.79	79	81	79.8	0.79	79	81
SNB	75.3	0.48	75	76	80.4	0.52	80	81
ANB	5	0	5	5	2.1	0.32	2	3
FMA	19.5	0.53	19	20	21.8	1.14	20	23
WITS	3.5	0.71	2	4	1.5	0.53	1	2
Saddle	128.9	0.88	128	130	125.1	0.88	123	126
L1NB	31.5	1.65	30	34	27.1	1.29	25	29
IMPA	100.3	1.06	99	102	94.7	0.48	94	95
U1NA	27.8	1.14	26	29	22.2	1.48	20	24
Lip Step	2.5	0.53	2	3	1	0.82	0	2
H angle	17.6	0.7	16	18	13	0.94	12	15

**Table 5:** Independent t test for comparison of the mean cephalometric values of the participants before and after treatment

**Variable**	**Assumption**	**t**	**df**	**Sig. (2-tailed)**	**Mean Difference**	**Std. Error Difference**	**95% CI Lower**	**95% CI Upper**
ANB	Equal variances assumed	29	18	0	2.9	0.1	2.68991	3.11009
ANB	Equal variances not assumed	29	9	0	2.9	0.1	2.67378	3.12622
FMA	Equal variances assumed	-5.811	18	0	-2.3	0.39581	-3.13157	-1.46843
FMA	Equal variances not assumed	-5.811	12.707	0	-2.3	0.39581	-3.15711	-1.44289
WITS	Equal variances assumed	7.171	18	0	2	0.27889	1.41408	2.58592
WITS	Equal variances not assumed	7.171	16.642	0	2	0.27889	1.41063	2.58937
Saddle	Equal variances assumed	9.704	18	0	3.8	0.39158	2.97733	4.62267
Saddle	Equal variances not assumed	9.704	18	0	3.8	0.39158	2.97733	4.62267
L1NB	Equal variances assumed	6.65	18	0	4.4	0.66165	3.00993	5.79007
L1NB	Equal variances not assumed	6.65	16.991	0	4.4	0.66165	3.00399	5.79601
IMPA	Equal variances assumed	15.21	18	0	5.6	0.36818	4.82649	6.37351
IMPA	Equal variances not assumed	15.21	12.587	0	5.6	0.36818	4.80194	6.39806
